# Links between mental health problems and future thinking from the perspective of adolescents with experience of depression and anxiety: a qualitative study

**DOI:** 10.1186/s13034-023-00679-8

**Published:** 2023-12-21

**Authors:** Peiyao Tang, Georgia Pavlopoulou, Katarzyna Kostyrka-Allchorne, Jacqueline Phillips-Owen, Edmund Sonuga-Barke

**Affiliations:** 1 School of Academic Psychiatry, Institute of Psychiatry, Psychology & Neuroscience (IoPPN), King’s College London, 16 De Crespigny Park, London, SE5 8AF UK; 2 Group for Research in Relationships and Neurodiversity (GRRAND), Clinical, Education & Health Psychology, Division of Psychology & Language Sciences, Faculty of Brain Sciences, University College London, London, UK; 3 Anna Freud Centre, London, UK; 4 Department of Psychology, School of Biological and Behavioural Sciences, Queen Mary University of London, London, UK; 5 Department of Child & Adolescent Psychiatry, Aarhus University, Aarhus, Denmark

**Keywords:** Depression, Anxiety, Adolescence, Future thinking, Prospection, Qualitative interview

## Abstract

**Background:**

Depression and anxiety are common during adolescence and could have detrimental impacts on young people’s ability to make and implement plans for their future. However, to the best of our knowledge, no other study has adopted a qualitative approach in investigating these effects from the perspective of adolescents with lived experiences of depression and anxiety. We sought to understand how young people perceive and interpret the impact of mental health conditions on their thinking about the future.

**Methods:**

We conducted semi-structured interviews with 19 adolescents aged 16–19 years in the UK (median age = 19, IQR = 1.5), who had a history of protracted periods of clinical or subclinical depression and/or anxiety. They were asked to reflect on how their ability to think about the future and the content of the future-related thinking was impacted during periods of poor mental health, compared with periods of feeling well. Interviews were transcribed verbatim and subjected to thematic content analysis.

**Results:**

Five domains were identified. First, *the impact of mood on future thinking capability* focuses on reduced ability and motivation to engage in future thinking. Second, *the impact of mood on images, thoughts, and feelings about the future* includes the emotional valence of future-related thoughts, their vividness, structure, and the extent to which they intimated subjective feelings of control (i.e., agency). Third, *social influences* focuses on social factors that might ameliorate or exacerbate future thinking. Fourth, *reflections on personal worries and expectations about the future* captures personal interpretations of past worries and hopes and how future thinking affected mood. Finally, *personal coping* refers to how young people cope with the negative emotions that come with future thinking.

**Conclusions:**

This study provided a nuanced and granular account of how depression and anxiety impacted young people’s future thinking based on their lived experiences. By highlighting the different ways that variations in future thinking were experienced as a function of depression and anxiety, our analysis highlighted new factors that should be considered in studies of adolescent mental health risk, which could inform the development of new therapeutic approaches.

## Introduction

Adolescence is a transitional period of development involving both personal growth and life challenges, when the decisions young people make can have life-long consequences [1, 2]. It is characterised by a combination of unique physiological and psychological needs [3], for example, identity formation and personal development, as well as the marked increase in the demands and pressures of the social context [4].

Adolescence is also a period of a rapid increase in mental health problems, especially depression and anxiety [5, 6]. These conditions can produce states of mind that impact the ability to mentally re-experience personal events from the past and imagine events that could happen in the future, termed episodic cognition [7]. Research shows that the amount and quality of episodic cognition, especially future-related, is connected with adolescents’ ability to make realistic and effective plans [8–12]. Therefore, depression and anxiety could potentially reduce adolescents’ capability to clearly imagine the future or alter the content, structure, and valence of future-related thoughts, and, consequently, reduce their ability and motivation to make future plans [13].

The existing literature focuses on the content of future thinking and its links with depression in adults. Adult depression is associated with hopelessness about the future, both concurrently and longitudinally [14–18]. Similarly, an optimistic future outlook predicts a lower risk of subsequent depression, while negative and pessimistic expectations are related to higher depression symptoms [19–22]. States of mind related to depression are characterised by a reduced motivation to act in relation to the future and generalised negative expectations of future events [23–26]. Depressed individuals have reduced feelings of control over their future, and also report a lack of motivation and confidence to make decisions in pursuit of their desired future goals [27].

Anxiety disorders have detrimental impacts on future-related thinking as well. Research shows that anxiety disorders are associated with a cognitive bias to threat [28]. Therefore, anxious adults are more likely to interpret ambiguous information about the future as threatening [29–33]. In addition, research demonstrates a negativity bias where anxious adults judge that negative future events are more likely to happen to them than neutral or positive ones [34]. Moreover, anxious individuals are also more specific when speculating about negative events than about positive events [35]. As a result, anxious adults tend to delay and avoid making decisions about their future due to their negatively biased interpretations of and increased vigilance to unpredictable future events [36, 37].

Existing studies examining the relationships between future thinking and mental health in late adolescence generally investigate questions similar to those asked in the adult literature. Therefore, studies tend to focus on generic aspects of future thinking, such as hope, optimism, pessimism, and future expectations, and how they relate to depressive and anxiety symptoms. Similar to findings from adult studies, hopelessness, negative future expectations and a lack of agency are found to be associated with subsequent depression and anxiety symptoms in late adolescence [38–48].

As adolescence is a unique and crucial developmental stage, the particular characteristics and needs of adolescents should be considered in research. Simply adopting the instruments developed for adults risks giving a limited picture of the links between mental health variations and future thinking in adolescence [49–51]. A study focusing on this developmental period is needed to explore and identify the unique patterns of future thinking and how it is linked with mental health problems during this time.

In this research, we took a necessary first step by undertaking a qualitative interview study with adolescents aged 16–19, who had experienced depression and/or anxiety, to explore the impact that poor mental health had on their future thinking at that time. The study specifically sought to answer three research questions: (1) How could pre-existing concepts of future thinking be enriched by perspectives of young people with a history of depression and/or anxiety? (2) How do young people describe future thinking during periods of poor and good mental health? (3) What do young people think about the link between future thinking and mood states? We did not set out to examine the themes that may be specific to either disorder due to their high comorbidity (i.e., depression and generalised anxiety; [52, 53]), but rather focused on exploring variations in the future thinking during the periods of poor mental health and in the well state.

## Methods

### Recruitment and participants

We adopted the qualitative approach, which was particularly well suited to the current study as it required adolescents to identify areas and issues in their own experience that may be missed if the study was based on other people’s accounts of adolescent experience or the adult-related literature more generally. We interviewed a community sample of adolescents, who had had a history of high depression and/or anxiety symptoms after age 12 but were managing well at the time of participating. This included either having a clinical diagnosis or subclinical elevated symptoms (self-reported) that had been present for three or more days in a typical week over at least three months. This approach enabled us to consider adolescents’ lived experiences of mental illnesses and to obtain their perspective at a time when they had developed clear insights to reflect upon their future thinking during a period of poor mental health.

The study was advertised via the university recruitment circular, social media and mental health charities. Adolescents who expressed an interest in the study were screened for moderate to severe depressive and anxiety symptoms using a questionnaire adapted from the Schedule for Affective Disorders and Schizophrenia (K-SADS-PL; [54]). The participants were not known to any of the authors prior to their participation, and participants were aware of the aim of this research and that it forms part of a PhD project in a UK institution.

The ethical approval was obtained from the Health Faculties Research Ethics Sub-committee at King’s College London (HR/DP-20/21-21394). All eligible participants provided written informed consent.

### The semi-structured topic guide

We identified and summarised six main concepts of future related thinking from the existing literature and structured the topic guide around these pre-specified concepts (Table 1). This was implemented flexibly with variations in the order and wording of the questions. Follow-up questions were added where appropriate to probe for more in-depth reflections and comments. The interviewer probed for a contrast of future thinking between periods of poor mental health and the well state throughout the session. The topic guide was pilot-tested with two adolescents (one male aged 19 years and one female aged 16 years), and questions were dropped or modified where necessary.

**Table 1:** The six concepts of future thinking covered in the topic guide

Concepts	Description of the concepts	Example questions
Capability	Whether young people can think about the future	‘Are you able to think of images of a future self? Can you describe what it’s like?’ ‘Has there been a time when you don’t feel like you have a future or don’t want to think about the future?’
Content	What the future thinking involves, if they can think about the future	‘When I say ‘the future’, what does it mean to you? What thoughts does it raise in your mind?’ ‘How do you think things are going to be in the future?’
Frequency	How often do they think about the future	‘How often do you imagine your future self?’ ‘Has there been a time when you think more or less about your future?’
Structure	Whether the thinking is focused/fixated on a single event or a series of events	‘Do you see a series of negative events?’ ‘Do you dwell on the same thing over and over again?’
Valence	Whether the content of their thinking is negative, positive, or neutral	‘How do you feel about the future?’ ‘Do you look forward to your future? What are you hoping for? Are you worried about your future?’ ‘Has the way you feel towards the past/future changed? Have you become more optimistic/pessimistic about the future?’
Salience/vividness	How clear the details and images of that thinking are	‘When you think about your future, do you think about concrete plans and specific situations?’ ‘When you think about your future, is it clear or quite blurry?’

### Procedure

All interviews were conducted remotely on Microsoft Teams and audio/video-recorded. Only the participant and the first author (PT, M.Sc., female) were present in the interviews. All participants were interviewed once in a single session. At the beginning of each interview, participants completed a demographic questionnaire that asked about their age, sex, geographic location, and education level. After the interview, participants were thanked and provided opportunities to ask questions. Each participant received a £10 shopping voucher as an appreciation for their time. Field notes were recorded after the interviews describing the researcher’s reflections on the conversation.

All interviews lasted between 25 and 43 min. The transcripts of the interviews were not returned to participants for comment or correction. Prior to the current study, the first author had previous experience of conducting interviews in another qualitative study and had then received training on qualitative research design, methods, data collection and data analysis.

### Coding and analysis

We analysed the data using thematic analysis [55, 56] with a blended inductive/deductive approach. The initial generation of the codes was influenced by the six pre-defined concepts of future thinking identified from the literature review of the existing future thinking measures and the aspects they capture (i.e., *Capability, Content, Frequency, Structure, Valence, and Salience/vividness*). The codes were then refined and modified using the interview data. We worked deductively starting with a few codes based on the pre-defined concepts (e.g., lack of *capability*, a blurry image for the future, and negative repetitive thoughts about the future), but then inductively adding up new codes and developed the codes iteratively as we reviewed the data.

We used thematic analysis to facilitate a flexible yet detailed interpretation of the dataset and a blended approach to facilitate consideration of the data in relation to Frost’s model [57], alongside new interpretations that arose during the coding process. We drew on a critical realist framework in our interpretations of the data [58] as it is a middle ground between positivism (concepts that are derived from scientific observations and predictions) and constructionism (subject truths that are constructed via interactions). This position allows for an observation of ‘reality’, whilst acknowledging that unobservable thoughts, perceptions and knowledge may have driven the observed events and is thought to be an appropriate approach to understanding a specific psychological phenomenon [59].

We referred to the COnsolidated criteria for REporting Qualitative research checklist (COREQ; [60]) and Yardley’s [61] criteria of good qualitative research in conducting and reporting the current study. Our study demonstrated *sensitivity to context* by showing awareness and openness of the participants’ perspectives and the setting. The researchers remained mindful that our positionality may have influenced the participants’ answers and that the identified codes and themes originated from the participants’ narratives. The multidisciplinary research team had expertise in qualitative research and adolescent mental health (GP), child and adolescent psychiatry (ESB, KA) and affective disorders (PT, JPO). Through data analysis iterations, we undertook reflexive dialogues on potential sources of bias and our prior assumptions that may come from personal, research, and professional experiences with the research topic to improve the awareness of our subjective influences on the research process.

Crucially, we were able to show *sensitivity to the data* by carefully considering the meanings generated by the participants. *Commitment and rigour* was also demonstrated by the in-depth engagement through data collection and undertaking a detailed, in-depth analysis. *Transparency* was achieved by reporting all steps and including tables of codes and quotes for the reader to clearly see how the interpretation was derived from the data.

Interviews were transcribed verbatim and de-identified as soon as they were completed, and the first author started coding when the first five interviews were done. Further interviews were coded as they were conducted in an iterative process, and the authors discussed and agreed on when data saturation had been achieved (i.e., when further interviews would not add new valuable insights [62]). We regularly cross-checked between the co-authors to make sure the codes do not reflect a single person’s interpretation alone. The first author conducted the initial coding and generation of the themes. All co-authors participated in checking and refining the themes and domains. Data were managed and analysed using NVivo (Lumivero NVivo, Version 13, 2020, R1).

## Results

### Participant characteristics

Nineteen participants (16 females) aged 16–19 years (mean = 18.2, standard deviation; SD = 1.2) were recruited in the UK. Eight (42%) participants were White, five (26%) were Asian/Asian British, four (21%) were Black/Black British, and two (11%) were mixed race. Thirteen (68%) participants had a clinical diagnosis of depression and/or an anxiety disorder, and six (32%) had subclinical self-reported symptoms. The time between the first depression/anxiety episode and the interview ranged from 6 months to 6 years (mean = 3.1, SD = 1.9). The demographic characteristics of participants are presented in Table 2.

**Table 2:** The demographic characteristics of participants

Participant number	Age	Gender	Ethnicity	Diagnosis
1	18	Male	Asian/Asian British—Indian	Clinical depression and anxiety
2	19	Female	Black/Black British—African	Depression and anxiety (self-report)
3	19	Female	Black/Black British—African	Clinical post-traumatic stress disorder
4	16	Female	Asian/Asian British—Indian	Clinical depression and anxiety
5	19	Female	White British	Clinical depression and anxiety
6	19	Female	White—Other	Depression and anxiety (self-report)
7	19	Female	White British	Clinical anxiety
8	19	Female	Asian/Asian British—Indian	Clinical depression
9	16	Male	Asian/Asian British—Vietnamese	Depression and anxiety (self-report)
10	19	Female	Black/Black British—African	Clinical post-traumatic stress disorder
11	18	Male	Black/Black British—African	Clinical depression and post-traumatic stress disorder
12	19	Female	Asian/Asian British—Indian	Depression and anxiety (self-report)
13	19	Female	White British	Clinical depression and anxiety
14	19	Female	White British	Depression and anxiety (self-report)
15	17	Female	White British	Depression and anxiety (self-report)
16	19	Female	Mixed—White British and Indian	Clinical depression
17	17	Female	White British	Clinical depression and anxiety
18	16	Female	Mixed—White British and Asian	Clinical mixed anxiety and depressive episodes
19	19	Female	White British	Clinical depression and anxiety

Five domains were identified: domain one, *the impact of mood on the future thinking capability;* domain two, *the impact of mood on images, thoughts, and feelings about the future;* domain three, *social influences;* domain four, *reflections on personal worries and expectations about the future*; and domain five, *personal coping*. Each of them includes two to four themes (for a list of themes, see Table 3).

**Table 3:** Overview of the coding structure

Domains	Themes
The impact of mood on the future thinking capability	*Varied ability and motivation*
	*Stuck in the past*
The impact of mood on images, thoughts, and feelings about the future	*Valence*
	*Vividness*
	*Agency*
	*Structure*
Social influences	*Social pressure and support from friends and peers*
	*Pressure from parents and financial hardship*
Reflections on personal worries and expectations about the future	*The impact of future thinking on mood*
	*Optimism, pessimism, and future expectations*
Personal coping	*Engaging in future thinking as a strategy to combat depression*
	*Scientific thinking, to challenge perception with evidence*

### Domain 1: The impact of mood on the future thinking capability

The concept of *Capability* refers to the quality of being able to think about the future. It includes the cognitive ability to engage in future thinking and the willingness and motivation to proactively think and plan for the future. Two themes were identified: *varied ability and motivation* and *stuck in the past* (see Table 4).

**Table 4:** Themes and quotes of *‘The impact of mood on the future thinking capability’* during poor mental health and well state

Themes	Poor mental health state	Wellness state
Varied ability and motivation	*“I think it was more of a lack of capability in the sense of not want, being so scared of it, that you don’t even want to acknowledge it. So I would avoid thinking about it because it would, one, make me more stressed, and two, make me more sad.” [06*^*1*^*; female, 19*^*2*^*, subclinical depression and anxiety]* *“I hated thinking about the future. I didn’t want to think. People at the time tried to help me a lot by saying, ‘but it won’t. You won’t always feel anxious. It’ll be OK in the future’, but I don’t want to think about that, because I didn’t think that the future would ever change. I thought it would always be like that.” [17; female, 17, clinical depression and anxiety]*	*“I can think a lot more further into the future without getting upset, and I guess I let myself have fun imagining things.” [18; female, 16, clinical mixed depression and anxiety episodes]* *“But now, I look forward to the future, I forget about the negativity, I don’t dwell on each day thinking about what’s happened. I’m just thinking about tomorrow. Tomorrow is going to be a good day.” [01; male, 18, clinical depression and anxiety]*
Stuck in the past	*“When I’m highly anxious and in that sort of state, I don’t really think of anything else apart from what’s happening right now. … I don’t know if it’s you don’t want to look at it [the future] or you just give up thinking about it cause you’re so highly stressed about the current state, you would inward looking rather than thinking about the future.” [07; female, 19, clinical anxiety]*	*“I’m still quite past orientated, but at the same time I’m a lot more future orientated. … I do look more into the future now, and that’s me actively trying to do that, cause I think sometimes when I get stressed out, I’m trying to think, well there are better things to come and they’ll be there soon.” [07; female, 19, clinical anxiety]*

^1^Participant number

^2^Age of the participant

#### Varied ability and motivation

Participants frequently talked about a lack of physical and mental capacity to think about the future when they were depressed and/or anxious. Some attributed it to being focused on and overwhelmed by what was going on at the time, leaving no ‘space’ to think about the future at all.

*“I was feeling like I couldn't go on any longer, cause it's feeling too low, too down. I was struggling so much that I couldn't think about the future. I knew the future was there, but I couldn't get out of the bubble.” [01; male, 18, clinical depression and anxiety]*

Others avoided thinking about the future, as they feared it would further worsen their mental health.

*“I would avoid thinking about it, because it would, one, make me more stressed, and two, make me more sad.” [06; female, 19, subclinical depression and anxiety]*

Some participants also described the time of poor mental health as ‘*passive living*’, in ‘*autopilot mode*’ and ‘*zombie working*’, where they followed routines and worked on what had to be done, without considering the consequences of their actions in the future.

*“I physically and emotionally couldn't imagine anything beyond. … It was more of a day-by-day thing, so it wasn't like five years into the future, not even a year, just literally taking each day at a time and trying to hold onto something each day.” [16; female, 19, clinical depression]*

#### Stuck in the past

During the poor mental health state, adolescents reported dwelling upon the past more than the future. They tended to think more about the events that had happened, especially those with negative consequences, such as their past mistakes. The future was often less considered or not thought about at all.

*“Whenever I thought about the future back then, it was never good. … What I thought would be only getting worse, never getting better. I would dwell upon the past; as for the future back then, I didn't even have an image.” [04; female, 16, clinical depression and anxiety]*

During the well state, adolescents were more future-oriented and thought more about the future than the past. A few participants generally became more reflective and introspective and thought more about both the past and the future.

*“I’ve learned that, forget about the past and move forward. I don't pay attention to what has happened because that's gone. You need to be thinking about tomorrow.” [01; male, 18, clinical depression and anxiety]*

*“I try to focus more on the future, but I also remember the past. The past has made me the person that I am.” [10; female, 19, clinical PTSD]*

### Domain 2: The impact of mood on images, thoughts, and feelings about the future

This domain captures the images, thoughts, reflections, and feelings adolescents had about the future and includes four themes: *valence*, *vividness*, *agency* and *structure* (see Table 5).

**Table 5:** Themes and quotes of *‘The impact of mood on images, thoughts, and feelings about the future’* during poor mental health and well state

Themes	Poor mental health state	Wellness state
Valence	*“It really affected me, because I felt like I just didn’t have any sense of purpose. I was so nervous about everything. I was even afraid everything will probably went wrong around me.” [10; female, 19, clinical PTSD]*	*“But now when you’re better after going through that [depression] and coming out, you plan about something. So you are really looking forward to achieving that goal that you have set in an exam for example.” [03; female, 19, clinical PTSD]*
Vividness	*“Blurry, definitely blurry. I didn’t know anything. I didn’t know what I wanted to do.” [04; female, 16, clinical depression and anxiety]*	*“Now I can think straight, I’m optimistic and I can think about the future. I’m more realistic in thinking about something than before, at least I can think about the steps towards achieving that thing, and I now know that there are a lot of challenges before achieving.” [11; male, 18, clinical PTSD]*
Agency	*“I had no control over what I was thinking about at that time. It just came. And slowly by slowly, I was losing control of everything.” [11; male, 18, clinical PTSD]*	*“It [the future] is all in my hand, so it depends how much work I put in, how much I actually really want it. Cause if I really want it, then I’ll make sure it comes true.” [04; female, 16, clinical depression and anxiety]*
Structure	*“I usually think of everything that could possibly go wrong and then just play it in my head. … It’s overwhelming because you just end up spiralling. There’s no positive way out, as soon as you start overthinking, you’ve almost set yourself up to spiral.” [14; female, 19, subclinical depression and anxiety]*	*“When I was in a really anxious day, I looked at very small things as being super long period of my life and being a massive event that was going to affect everything else. I’ve come to realise a little bit better that actually those small events are just really small in the whole span of my timeline of life. So I look to the future with more possibilities, and there’s greater time.” [07; female, 19, clinical anxiety]*

#### Valence

During poor mental health states, adolescents’ future thinking was predominantly negative. Participants reflected that when they had been depressed and anxious, they had been irrationally negative about the future, afraid of academic failure, failing their own and others’ expectations and never feeling good enough.

*“Mainly negative, I don't remember thinking anything positive.” [05; female, 19, clinical depression and anxiety]*

During the well state, adolescents were generally hopeful for the future. They believed their expectations and hopes for the future would come true.

*“I used to think that the future will also be bad. So like goals, targets, I never used to have those, just living for the moment. But right now, I've set up goals and targets, and I'm actually hopeful for the future and how it will be.” [02; female, 19, subclinical depression and anxiety]*

#### Vividness

During the poor mental health states, participants described a disconnection with their positive future selves. Some were unable to visualise the future at all. Others described it in terms of ‘black, blank and blurry’ images. In contrast, they had vivid images of a negative future self and their fears and worries about the future were described in rich detail, which even felt like reality.

*“Cause I was so negative being in depression, it just became like there was no light at the end of the tunnel. It was so blurred in the whole process that I couldn't think what am I moving towards?” [01; male, 18, clinical depression and anxiety]*

*“I could see myself just not having done well, coming back home and having to answer questions, just staying in my room and crying, because I couldn’t do it. It was more vivid than other images of the future.” [08; female, 19, clinical depression]*

During the well state, adolescents saw their future in more detail and specificity and described more connections to a positive future self. They were able to generate clear images of a future self and speculate their future targets and plans to achieve them.

*“I've got a lot of things that I look forward to. I'm always thinking one step ahead, about third year, and what speciality, what skills.” [05; female, 19, clinical depression and anxiety]*

#### Agency

During poor mental health states, some participants described a lack of agency, where they did not feel in control of their future or their negative feelings. Some described it as being externally controlled, for instance, ‘*a ghost or spiritual curse*’, ‘*a religious punishment*’, and ‘*a monster in my head*’.

*“Interviewer: Do you think you have control over the future?*

*Participant: No, not at all. … I felt like there was a huge lack of control, and I felt that in terms of every aspect. … That lack of control was the most frustrating part because it's dehumanising in a way. Every aspect of life, like appetite, sleeping, friends, walking, was affected.” [19; female, 19, clinical depression and anxiety]*

In contrast, in the well state, adolescents felt more control of their future and automatic negative thoughts. They became more flexible with planning for the future and accepted that some of it was beyond their control. They were also able to see a longer-term and larger-scale future instead of overthinking the small things right in front of them. They used evidence and scientific thinking to combat worries and were able to distinguish perception from reality.

*“I can't think of anything else that would play a bigger part than my own choices and my own vision.” [08; female, 19, clinical depression]*

#### Structure

When thinking about the fears and worries for the future during the poor mental health state, participants described ruminating on and being with their thoughts in negative cycles. They worried that a single negative event would lead to a series of subsequent events or something even worse (serial thinking). They also felt negativity would be enduring while positivity was only temporary by constantly playing down their achievements and successes.

*“I felt so out of control with my emotions that I felt like it was going to be like that forever. I would think, I've been like this for a month and a half, it's never gonna end, I'm gonna continue like this for the rest of my life.” [06; female, 19, subclinical depression and anxiety]*

When well, some participants were able to shift their focus from negative fixated thoughts about minor details. This allowed them to let go of the negativities and regrets and free themselves from the vicious cycle of being caught up in rumination. Some described this as ‘*feeling more focused again on what’s important*’.

*“I used to be so focused on overthinking the thing that was gonna come up. Now I can look at more things, at the bigger picture, so my whole life perspective, sequencing and timeline.” [05; female, 19, clinical depression and anxiety]*

#### Domain 3: Social influences

The *Social influences* domain refers to the direct and indirect influences from young people’s family and peers on their future thinking. The social context has both negative and positive influences on participants’ future thinking. This domain includes two themes:* social pressure and support from friends and peers*, and *pressure from parents and financial hardship* (Table 6).

**Table 6:** Themes and quotes of *‘Social influences’*

Themes	Quotes
Social pressure and support from friends and peers	*“I definitely think my friends were the most positive thing about that time, and because they made me feel so valued, I thought I have hope with my friends, like we’re gonna stay together even throughout this, even after everything. And my sister, she definitely kept hope in me about college, just holding on even after GCSEs.” [04; female, 16, clinical depression and anxiety]*
Pressure from parents and financial hardship	*“I think the fact that you’ve grown up now, and also my parents don’t provide everything for me. You have to look for a job, and you have to plan for yourself and your lifestyle. So through that, it just comes automatically, you just have to plan for the next day.” [03; female, 19, clinical PTSD]*

#### Social pressure and support from friends and peers

Talking about the future with friends and peers could bring fear and anxiety. Social pressures could exacerbate the anxiety about one’s future.

*“There are those people who believe in you, and they believe you can really do it, but as always, we have people who’re so negative and they discourage you in everything. … Why do you think you are special? People failed before, why would you succeed?” [11; male, 18, clinical depression and PTSD]*

However, the influences from friends and peers were described positively as well. Engaging other people in thinking about the future provided encouragement and inspiration and brought hope and excitement. Participants talked about having seen people close to them recover from depression and achieve their hoped-for future through hard work, which helped them feel more optimistic. Social support throughout depressive/anxiety periods also brought hope about the future.

*“It’s a good inspiration, when I saw that person got out of depression, worked hard and succeeded, it motivated me to concentrate in my dreams and goals, and just stop getting sad most of the time. So I thought even my life can be that way. I had a more optimistic way of thinking.” [02; female, 19, subclinical depression and anxiety]*

#### Pressure from parents and financial hardship

Family and parents made future thinking stressful for adolescents when the future they wanted for themselves deviated from the one their parents wanted them to have. This also made adolescents feel they lacked control over their future.

*“My education wise, at that time, they [my parents] were making all my decisions for me. That’s why I felt like I had zero control of my future, and that whole thought process would spiral in my head all the time. I wouldn’t have considered [the future], because everything was done, all the decisions were made.” [04; female, 16, clinical depression and anxiety]*

Growing up in a socioeconomically disadvantaged household also created more pressure to think about the future, as these adolescents had to plan how to create a more positive future for themselves.

*“I think what worries me is money, if I’m not able to get a good job, to pay off my bills, to live the expectations of life I’m looking forward to, because I haven’t had that in life, I come from a deprived neighbourhood. I don’t wanna be on benefits. I wanna do something in life, I hope that it is a positive future ahead for myself.” [01; male, 18, clinical depression and anxiety]*

### Domain 4: Reflections on personal worries and expectations about the future

This domain captures participants’ reflections and interpretations about their past worries and hopes, and how different styles of future thinking affected their mood. It includes two themes: *the impact of Future thinking on mood*; and *optimism, pessimism, and future expectations* (Table 7).

**Table 7:** Themes and quotes of ‘Reflections on personal worries and expectations about the future’

Themes	Quotes
The impact of Future thinking on mood	*“Thinking about the future would only make my anxiety high, and make me want to just not go on anymore, so thinking about the future was just not a good idea. It didn’t even feel like I had, in my present moment, thought it out, so I don’t even think about the future.” [04; female, 16, clinical depression and anxiety]* *“I think now my relationship with the future is, I wouldn’t say it’s very good, I think it’s fine. I’m coping with it well and it doesn’t scare me to think about what I might be doing in two years or three years from now. I have an idea of what I want to do, I don’t know how to get there yet, but I know that eventually I will find the way.” [06; female, 19, subclinical depression and anxiety]*
Optimism, pessimism, and future expectations	*“I wanted to go to the College of my choice, not the College of my parents’ choice, and the subjects of my choice, so non-medicine subjects and subjects I was actually interested in, considering I have to spend two years studying them. These are all expectations that seemed unrealistic at the time, but now I’m actually doing it all.” [04; female, 16, clinical depression and anxiety]* *“Basically everything that I ever worried about happening didn’t happen. I can’t think of one thing that did happen.” [17; female, 17, clinical depression and anxiety]*

#### The impact of future thinking on mood

Although the relationship between future thinking and mood can be bi-directional, participants said that there was a stronger impact of future thinking on mood than the other way around. However, the relationship was not straightforward. Future thinking could cause negativity and anxiety when they were in a depressed/anxious state, and even thinking positively about the future did not improve their mood. However, the participant who engaged in future thinking as a strategy to remain hopeful recalled it having a positive impact *[19]*. In the well state, there was generally less influence of future thinking on adolescents’ mood, as one participant said:

*“I can think a lot further into the future without getting upset, and I guess I let myself have fun imagining things.” [18; female, 16, clinical mixed depression and anxiety episodes].*

#### Optimism, pessimism, and future expectations

When reflecting on periods of depression and anxiety, participants talked about how surprised they were when their ‘unrealistic’ positive expectations came true, and how worries they firmly believed would come true did not. Participants looked back at how they had underestimated their possible future achievements and overestimated the chance of failure:

*“Initially I thought I’d do really bad in my GCSEs, and that didn’t really come true. I thought I’d probably do quite bad in my A-levels, and I did better than I thought I would. And I thought I’d get to university, but I’ve done better than I thought I would.” [05; female, 19, clinical depression and anxiety].*

They also reflected on over-worrying and overthinking in general and how they subsequently learnt from these reflections to combat current worries and fears. They became more conscious and aware that they may have exaggerated their chance of failure. As a result, they were able to identify over-worrying more sensitively and challenge those thoughts with past experiences:

*“I’d tell myself that, looking back, I had nothing to worry about, it was all self-inflicted.” [04; female, 16, clinical depression and anxiety]*

*“I think that right now, I’m doing a lot better, and I’m seeing it like a learning experience because now I look back at it, I can analyse myself the way I felt, in order for me to avoid going back to that hole in the future.” [06; female, 19, subclinical depression and anxiety]*

### Domain 5: Personal coping

*Personal coping* refers to how young people cope with the negative emotions that come with future thinking. It includes two themes: *engaging in future thinking as a strategy to combat depression*; and *scientific thinking, to challenge perception with evidence* (Table 8).

**Table 8:** Themes and quotes of *‘Personal coping’*

Themes	Quotes
Engaging in future thinking as a strategy to combat depression	*“Imagine I meant to get from point A to B, I would stress a lot about GETTING to point B and not about the process of getting there, but now I’m just trying to enjoy the process of getting to point B. … I think it has allowed me to take things slow, and not want to rush to get to a specific place. Before, I would get more stressed if I didn’t get specific things done on time. Now, I’m a lot calmer about it.” [06; female, 19, subclinical depression and anxiety]*
Scientific thinking, to challenge perception with evidence	*“I’m a lot more scientific in my thinking in terms of basing it on evidence. I try to combat any worries and fears I have with what I have in front of me, so exams, for example, I’m starting to worry about my exam and I’m like, ‘what if I fail?’ But then I look at all the work I’ve done, and all the revisions and all the plans I’ve made for it, and I’m like, ‘well, there’s still a possibility it’s more unlikely’, to use that as a reassurance.” [05; female, 19, clinical depression and anxiety]*

#### Engaging in future thinking as a strategy to combat depression

When comparing future thinking during the poor mental health and well states, participants talked about being able to focus more on the future and less on worries and fears during well periods. They could plan the future more flexibly and look at the bigger picture instead of fixating on recurring negative thoughts, being rigid about set plans and rejecting the alternatives. They also acknowledged that some things were out of their control and allowed for more freedom visions for the future.

*“I realise how things can change in an instant, and it does impact how I think about the future in that, I try not to be as rigid with ‘this must happen, if I don’t get this job, I’m not going to be happy’. It also makes me realise that things happen and we don’t have that much control over them.” [07; female, 19, clinical anxiety]*

Alongside diverting the focus from the past to the future, there was also more focus on the present, enjoying the moment, taking things slowly, emphasising the process more than the outcome, and a generally more flexible approach to planning what they wanted to achieve.

*“It has allowed me to take things slow and not want to rush to get to a specific place. … Before, I would get more stressed if I didn’t get specific things done on time. Now I’m a lot calmer about it.” [06; female, 19, subclinical depression and anxiety]*

Among the 19 participants interviewed, one participant *[09; male, age 16, subclinical depression and anxiety symptoms]* stood out. Unlike the other adolescents who lost the motivation and capability to think about their personal future when depressed, he actively engaged in future thinking as a strategy to deal with his depressive symptoms and had vivid imagery of his future self throughout poor mental health periods.

*“There's a song, by BTS called Pied Piper. … So during my really depressing period, I would just listen to that song, and it kept me going. Even if I felt bad now, I knew it would end so that I could get into that future state. … So me thinking about bad stuff from the past or from now, I thought about the future to counteract it, to give me hope in that times.”*

Whilst other participants described a disconnection with a positive future self and an inability to visualise images of the future self, he had worked to create a detailed and positive future outlook during poor mental health periods. He intentionally engaged in future thinking, especially the pictures of a positive hoped-for future self, to protect himself against low mood and improve his motivation and confidence to achieve the ideal future self.

*“The detail of me being a mathematician in the future is extremely detailed. It’s me in front of a blackboard, with my hot chocolate and marshmallows, drinking, doing the problem on the board, just thinking really hard.”*

#### Scientific thinking to challenge perception with evidence

Participants also actively used strategies to manage and combat automatic negative thoughts by facing and dealing with them rather than avoiding them. Participants talked about engaging in scientific thinking, by challenging their perception with evidence rather than falling into the ruminative cycle.

*“I’m much better at analysing these thoughts and thinking, do they make sense? Are they logical or are they just something I don’t really need to worry about and I need to distract myself from?” [18; female, 16, clinical mixed depression and anxiety episodes]*

It should be noted that adolescents in this study had either depression and/or anxiety symptoms, or both, in the past. Although the study did not set out to distinguish between the two, one participant explicitly commented on the distinct impact that depression and anxiety had on her *[19; female, 19, clinical depression and generalised anxiety disorder]*. She described not thinking about the future at all during depression, while anxiety being the opposite, overthinking and overanalysing the future:

*“So for depression, it’s avoidance, denial to see the future; and anxiety, it’s overanalysing, overthinking and also irrational thoughts. Depression is very different in the way that the future is not even a consideration; anxiety is the polar opposite, it’s on your mind, it’s intrusive, it’s over control, like overpowering.”*

## Discussion

In this study, we aimed to explore the views of adolescents who had experienced depression and anxiety about how poor mental health impacted their future thinking and vice versa. We adopted a qualitative approach and identified key domains and themes in which future-related thinking was either influenced by or contributed to depressive and anxious symptoms.

First, we identified a lack of ability *(not being able)* and motivation *(not wanting)* to think about the future during periods of poor mental health, as well as a stronger orientation towards the past over the future. Some participants suggested that together, the cognitive inability and emotional avoidance of thinking about the future may have prevented depression and anxiety from getting worse (*“because it would, one, make me more stressed, and two, make me more sad.”* [06]), as well as being a consequence of the low mood (*“depression really hit me hard, I was mostly just zombie-working.”* [11]). Previous studies have also reported the loss of motivation and struggles with maintaining and actively engaging with long-term goals. They have demonstrated that depressed adolescents have blunted neural responses in their anticipation and efforts to gain reward and avoid punishers [63–65]. Few existing studies specifically investigated the lack of ability and motivation to engage in future thinking and mostly quantified the ability of future thinking in relation to the time needed to generate future events or the clarity of elaborated future events [51, 66–70]. Moreover, in this study, we found that young people were predominantly past-oriented during a depressed and anxious state, which is in line with existing research that depression is associated with an increased orientation towards the past and an avoidance of the future [71–73].

Second, young people’s perspectives of what their future thinking was like during depressive and anxious episodes presented a broad picture of its content, including the *valence*, *vividness*, *structure*, and young people’s *perceived sense of control*. The thoughts related to the future were predominantly negative, which is consistent with previous research that depression and anxiety are related to an increased generation of negative future events [34, 74]. It is worth noting that apart from increased negative emotions towards the future, some studies have also reported an absence of positive emotions or any emotions at all, which leads to feeling empty, dull, and blank [75–77]. This was captured in our findings of *vividness*, where young people described a disconnection with a positive future self and consequently, having a blank image of the future and losing track of their goals. However, as one participant *[19]* described, future thinking in anxiety is characterised by heightened worries and repetitive negative anticipation of future events and is completely different compared with depression [35]. The future may be perceived as closer than the past in anxiety, whereas the opposite may be the case in depression, although only one participant in our study elaborated on this point [78]. Furthermore, poor mental health state was related to a shift of focus from the bigger picture of the future to a fixation on small details (captured by *structure*), possibly reflecting a belief that the negativity will be constant and they will be ‘stuck’ in this state forever, which aligns with the *loss of a wider perspective* reported in Watson et al. [65].

We found mixed positive and negative influences of the social context on young people’s future thinking, where social support and pressure from significant others, including friends, peers, and parents, can both encourage and hinder their future thinking. It is worth noting that when talking about the support and encouragement from people around them, more young people referred to friends and peers than family members. In line with the study of Sica et al. [79], peer support possibly provides a beneficial effect on strengthening engagement with one’s future in early adulthood. However, for young people who are already indifferent and avoidant of future thinking, influences from peers may deepen their avoidance of the future and consequently hinder future planning. In our study, participants coming from a lower socioeconomic background reported having more pressure to plan for their own future and more worries about whether they would be able to live a life of their expected standard. This was consistent with findings from Fakkel et al. [80] that disadvantaged socioeconomic status is related to lower parental support and fewer positive future orientations.

On a more positive note, young people in our study reported having developed techniques and skills to adapt their future thinking and challenge negative repetitive thoughts from their experiences of past episodes of depression and anxiety. On reflection, they reported that their past worries and fears built up the strength and resilience to cope with new challenges of the future, allowing for increased flexibility with their future planning. This is particularly important given that existing research provides evidence for the protective effect of resilience, flexibility, and positive affect on a lessening of self-reported depressive symptoms and a stable remission from clinical depression in adults [81, 82]. Interestingly, participants reported a stronger influence from future thinking on mood than the other way around, while also acknowledging the impairing impacts depression and anxiety had on their future thinking. The dynamic pathway between future thinking and mental health needs to be further examined regarding the directionality and temporality.

### Strengths and limitations

To our knowledge, this is the first qualitative study to explore adolescents’ views on their future thinking abilities and patterns and how these are related to depression and anxiety. Compared with previous studies which examined separate future-related thinking aspects, we provided a more comprehensive perspective on the role future thinking plays in adolescent mental health. We explored aspects of future thinking that may originate from or exacerbate poor mental health from the accounts of adolescents with lived experiences of clinical or sub-clinical depression and anxiety. Furthermore, our study allowed the freedom to divert away from the adult models so that we could adopt a more developmentally appropriate approach.

One strength of the study was that we recruited participants of different ethnicities, varied socioeconomic backgrounds, education levels and geographic locations in the UK. However, there might still be a selection bias as participants have to be currently managing well to be deemed eligible. We were therefore unable to explore future thinking among those who were experiencing depression or anxiety at the time of participation. Our eligibility criteria were based on the consideration that adolescents who are unwell might not have the capability or motivation to reflect on their future thinking. Also, a few sensitive questions in the topic guide might elicit concerns for their mental health and safety.

Including both clinical and subclinical symptoms allowed us to investigate lived experiences of depression and anxiety on a continuum rather than a dichotomy, which provides a good representation of experiences in real life. However, we should also acknowledge the challenges of relying on self-reports here. For example, the participant *[09]* who reported still having a positive future outlook and used it as a coping strategy to combat depression may have been in a state of poor mental health, rather than a state of mental illness. Therefore, whether a self-reported depressive episode could be interpreted as being mentally ill should be taken into account when interpreting the study’s findings. However, about two-thirds of participants reported having clinically diagnosed depression and/or anxiety and their reflections did not appear qualitatively different from those without a diagnosis. Finally, although the findings are based on retrospective accounts, we included adolescents who experienced depression and anxiety after age 12, their experiences therefore were not distant temporally.

We did not seek to distinguish between future thinking patterns that were unique to depression and anxiety, as the comorbidity rates for these disorders are high in adolescence (e.g., between 15.9 and 61.9%; [52, 53]). Depression and anxiety symptoms are also highly correlated, making it challenging to identify young people with only depression and not anxiety, and vice versa. According to some participants, it would be hard for them to distinguish between the influences of depression and those of anxiety on their future thinking.

Despite the important insights provided by the qualitative approach in terms of understanding personal accounts and interpretations, it has limitations too. The findings are based on the individuals’ subjective experiences and are context-specific. This means that the generalisability of the findings to a wider population is limited. However, this research constitutes a preparatory stage of a longitudinal project on adolescent mental health and future thinking. The coding and analysis of the interviews informed the development of a new quantitative measure specifically designed for adolescent mental health and future thinking. Participants were shown the candidate items for the new measure and provided feedback in a separate session (for a detailed explanation, see Tang et al. [83]). By identifying aspects of future thinking that could present as either potential risks for or consequences of poor mental health, findings from the present study inform the design of a longitudinal phase of the project.

### Implications and next steps

From a clinical and practical perspective, this study demonstrated the profound impact that poor mental health has on adolescent decision-making and future planning, which was reflected in the narratives of participants. The findings emphasised the importance of supporting adolescents at the crucial stage of personal growth and life challenges. Given the relationship between poor mental health and reduced capability to engage in future thinking, the loss of intrinsic motivation and an overly pessimistic expectation may hinder young people from living up to their full potential. Therefore, young people who suffer from low mood and anxiety may need additional support to set up realistic, concrete and achievable future plans. Moreover, understanding the directionality between changes in future thinking patterns and adolescent mental health problems could provide important insight into identifying at-risk individuals and incorporating support with future thinking aspects in therapeutic approaches, such as promoting positive mental imagery and specificity in episodic future thinking, which were proven effective in treating clinical depression [84–88].

This study also extended the existing academic knowledge by providing a comprehensive and developmentally sensitive insight into a conceptual structure of future thinking in adolescent mental health problems. We identified future thinking patterns that are associated with depression and anxiety disorders in late adolescence, based on young people’s lived experiences. However, the causal pathways between future thinking and depression are yet to be determined. Although this was an exploratory qualitative study, it highlighted the factors to be measured in future quantitative studies, which could statistically test hypotheses about the direction and temporality of changes in future thinking patterns and mental health symptom severity.

## Conclusions

We explored the perspectives of adolescents with lived experiences of depression and anxiety disorders regarding how their future thinking was affected by poor mental health, compared with when they manage well. Depression and anxiety affected the ability and motivation to engage in future thinking and the valence, vividness, structure, and agency concerning future-related thoughts. Young people’s reflections on their past worries, fears, and expectations revealed how they learnt through the process of better coping with poor mental health and their personal interpretations of the interrelated dynamics between mental health and future thinking. Findings from this study provided evidence that young people who had experienced poor mental health felt that it had profoundly impairing effects on their future thinking across multiple aspects. It also highlighted the importance of studying the temporal relationships between episodes of mental health problems and future-related thinking patterns to clarify potential causalities.

## Data Availability

The first author had full access to data used in the study. The data of this study with identifiable information removed are available on reasonable request from the corresponding author. The data are not publicly available due to ethical restrictions.
